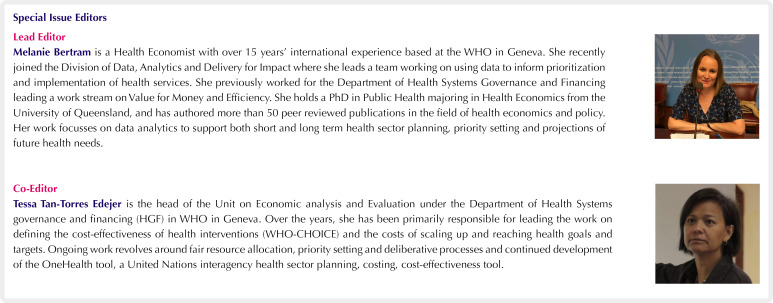# Introduction to the Special Issue on "The World Health Organization Choosing Interventions That Are Cost-Effective (WHO-CHOICE) Update"

**DOI:** 10.34172/ijhpm.2021.105

**Published:** 2021-09-19

**Authors:** Melanie Y. Bertram, Tessa Tan Torres Edejer

**Affiliations:** Department of Health Systems Governance and Financing, World Health Organization, Geneva, Switzerland.

**Keywords:** Cost-Effectiveness Analysis, Universal Health Coverage, Benefit Package, Economics

## Abstract

The WHO-CHOICE (World Health Organization CHOosing Interventions that are Cost-Effective) approach is unique in the global health landscape, as it takes a "generalized" approach to cost-effectiveness analysis (CEA) that can be seen as a quantitative assessment of current and future efficiency within a health system. CEA is a critical contribution to the process of priority setting and decision-making in healthcare, contributing to deliberative dialogue processes to select services to be funded. WHO-CHOICE provides regional level estimates of cost-effectiveness, along with tools to support country level analyses. This series provides an update to the methodological approach used in WHO-CHOICE and presents updated cost-effectiveness estimates for 479 interventions. Five papers are presented, the first focusing on methodological updates, followed by three results papers on maternal, newborn and child health; HIV, tuberculosis and malaria; and non-communicable diseases and mental health. The final paper presents a set of example universal health coverage (UHC) benefit packages selected through only a value for money lens, showing that all disease areas have interventions which can fall on the efficiency frontier. Critical for all countries is institutionalizing decision-making processes. A UHC benefit package should not be static, as the countries needs and ability to pay change over time. Decisions will need to be continually revised and new interventions added to health benefit packages. This is a vital component of progressive realization, as the package is expanded over time. Developing an institutionalized process ensures this can be done consistently, fairly, and transparently, to ensure an equitable path to UHC.

Around the world, all countries are working toward achieving the Sustainable Development Goals, as agreed at the United Nations General Assembly in 2015.^1^ Universal health coverage (UHC) is at the core of Sustainable Development Goal 3, aiming for health and wellbeing for all at all ages.

To achieve UHC, countries must focus on both progressive universalism and progressive realization, whereby the aim first to cover the whole population with high priority services, and after this over time expand the package of services available.^2^ This approach requires that a package of services that is affordable and can be guaranteed for all people without exposing them to financial hardship is established. This is known as the UHC Benefit or Services Package. The aim of health benefit package selection processes is to be consistent across all healthcare programmes and possible interventions, to ensure comparability and fairness in decisions made across the sector.

Cost-effectiveness analysis (CEA) is one criterion amongst many that can be used to set priorities and establish the UHC benefit package. Whilst the criteria chosen for selection are country specific and based on local values, commonalities tend to be seen across countries, with cost-effectiveness, budget impact, equity, feasibility and financial risk protection being regularly considered.^3^

WHO-CHOICE (World Health Organization CHOosing Interventions that are Cost-Effective) was established in 1998 to support priority setting and decision-making through the use of methodologically consistent cost-effectiveness ratios.^4^ The aim was to undertake priority setting exercises at the sectoral level, meaning across all diseases, in acknowledgement that all selection decisions come with an opportunity cost from the same pot of funds, therefore all disease areas must be considered simultaneously and with a consistent methodology.

Since the inception of WHO-CHOICE, the literature on the cost-effectiveness of interventions and services in low- and middle-income countries has increased significantly, yet it is still limited in terms of coverage of disease burden areas.^5^ To support countries in developing health benefit packages in settings where data are limited, WHO-CHOICE aims to provide tools for countries to use to estimate cost-effectiveness ratios in their local setting, as well as providing a global knowledge base of average and incremental cost-effectiveness ratios.

This series of papers presents a complete sectoral analysis using the WHO-CHOICE generalized CEA framework for the first time. Methodological updates are first presented,^6^ followed by sets of detailed CEA results for maternal, newborn and child health,^7^ major communicable diseases,^8^ and non-communicable diseases and mental health,^9^ before bringing these all together into examples of expansion paths signifying the most cost-effective packages of interventions.^10^

 The WHO-CHOICE series presents estimates for 479 interventions across 20 disease areas, but does not pre-define which of these interventions should be selected within an UHC benefit package. Results are presented identifying order of magnitude changes in average cost-effectiveness ratios, to support the identification of groups of interventions that might be considered cost-effective in different settings, depending on the available budget.

Across the three articles presenting new average cost-effectiveness ratio values,^7-9^ a consistent theme emerges that many WHO technical recommendations for clinical services are highly cost-effective in the two regions studied. Most of these highly-cost effective interventions are well-proven, low cost medicines where the predominant expenditure required is on human resources. Some interventions remain persistently less cost-effective than others, such as diabetes treatment, where high pharmaceutical prices endure.

The sectoral analysis presented in the final paper in the series is our first attempt to present comprehensive cost-effectiveness data across disease areas.^10^ The use of the common WHO-CHOICE methodology allows us to do so in a fair way, and avoids many of the known issues in comparability of CEA estimates, such as different comparators, study perspectives and discount rates.^11^

Of course, cost-effectiveness evidence alone is insufficient for developing a UHC benefit package, and the series authors are strong supporters of strengthening decision-making processes across all countries.^12^ Creating an evidence-based transparent and legitimate process for health benefit package selection, underpinned by a strong legal framework is crucial to fair decision-making for UHC. The processes of health benefit package selections are underpinned by the ethical framework of proceduralism, using the accountability for reasonableness framework and developing strong deliberative processes.^13,14^ WHO-CHOICE CEA estimates are intended for use alongside guidance documents advocating for transparent, participatory approaches to decision-making produced by WHO and others.^2,3,15,16^

At present WHO-CHOICE contains only a limited set of interventions, which reflect WHO guidance and focuses on a core set of interventions for UHC. Through the new Universal Health Coverage Compendium of Interventions (https://www.who.int/universal-health-coverage/compendium) WHO is working to expand the set of services included in benefit package discussions. In addition to this the Decide Hub, a global health network on Value for Money hosted at WHO (https://decidehealth.world/), is developing tools to support transferability of cost-effectiveness estimates so that the global literature can be used with consistency to support country decision-making processes.

Critical for all countries is institutionalizing decision-making processes.^16^ A UHC benefit package should not be static, as the countries needs and ability to pay change over time. Decisions will need to be continually revised and new interventions added to health benefit packages. This is a vital component of progressive realization, as the package is expanded over time. Developing an institutionalized process ensures this can be done consistently, fairly, and transparently, to ensure an equitable path to UHC.

## Ethical issues

 Not applicable.

## Competing interests

 Authors declare that they have no competing interests.

## Authors’ contributions

 MB and TE conceptualised the paper, MB wrote the first draft and TE provided critical review.

**Figure:**